# “Barsati,” or Atrophic Carcinoma

**Published:** 1887-04

**Authors:** Richard W. Burke

**Affiliations:** Cawnpore, India


### Art. XVIII.—BARSATI, OR ATROPHIC CARCINOMA.

BY RICHARD W. BURKE, A. D. V.

*Cawnpore, India.*

(Continued from page 66.)

I propose to call attention to the consideration of the more salient particulars concerning the clinical characteristics of this disease, which, though discussed nearly fifty years ago, are by no means an already completed work, but requiring greatly to be extended. With the facts recorded in the past regarding Barsati, it behooves both scientists and practitioners to look to their diagnosis, to improve if possible our knowledge of the clinical history of the disease, so that its application to pathology should become both wider and more precise; for the precision of our results in practice must depend upon the perfection of our diagnosis. There is a wide field before us in the study of this disease, and I trust others will join in ensuring its cultivation by extending their inquiries to every department of this important subject. The combined clinical experience of the veterinarians of the past fifty years or more, as recorded in the February and April numbers of the *Veterinary Journal* for 1885, is of great importance, and indicates many questions which urgently need answering. The relation of Barsati to cancer of man has presented great interest since our first announcement on the subject, and if the identity was not before actually suspected, the clinical observations and experiments which appear in the past literature of the disease clearly point in this direction, and which establish the identity contended by me beyond cavil or dispute. There are included in the facts given, reasons which, not refuted by the parasitic theory, require separate and permanent recognition.

We must now examine the several clinical features of Barsati and human cancer, and see if we can find that any of them show marked and definite relations to each other. I give quotations of the disease as seen in the two different species, for it is necessary to form a judgment as to the relative characters of each and to have a wide overlook on experiences which have been in the past recorded. My opinion is, and it is founded on careful investigations of many years, during which time I have witnessed the disease in its most varied and pronounced aspects, that Barsati is a disease common to robust condition of body, and that, although appearing in all animals, it is most frequently seen in horses having reputed good condition of body, the health only suffering in proportion to the advance of the disease from local parts to the internal organs. I believe the recorded experience of other veterinary surgeons shows this conclusively. And the experience of surgeons regarding cancer in man is, that, "prior to, and for a long time after the appearance of a cancerous sore, the constitution is distinguished, in most cases, by its remarkably healthy characters, and by the absence of any appearances which would indicate disease."* The great question which is thus raised is as to the condition of patients previous to the appearance of this disease, and the general answer is, "Remarkably healthy." Mr. Smith † characterizes the condition of horses subject to Barsati as "vigorous."

Most authors who have written on cancer state that simple wounds and ulcers sometimes become the seat of cancerous disease, and that the epithelial form of cancer does frequently arise in the chronic ulcers themselves. Now, what is the experience in regard to Barsati? I shall quote Mr. Hart,¶ who writes, "Simple sores and galls, if neglected, are very apt to assume *bursattic* action": which is the general experience of practitioners in India. Writers on cancer lay great stress on

*Treatise on Cancer, by R. Mitchell, 1879.
†Veterinary Journal, 1881, p. 308.
¶Veterinarian, 1873, p. 17.

"induration of the sore" as especially distinguishing cancer, a fact emphasized by those who have seen anything of Barsati before writing upon it. I. V. S. Oliphant,‡ in charge of Hapur Stud, observes: "From rubbing or biting—for the sore is particularly itchy—the crown of the ulcer becomes violently detached, leaving a moist, irritable sore, *the base of which speedily becomes indurated.*" Mr. Hart, in his paper on Barsati, describes "an indurated base extending to a depth of half an inch or an inch." Another important feature of Barsati, is the presence in the sores of pain or irritation felt at different intervals. Mr. Hart writes, what must be in the experience of every one, that, "Barsati sites, though healed, are liable at any time to become irritable and to be gnawed by the horse; this act showing beyond dispute that some irritative action is going on." This pain is certainly considered very characteristic also of human cancer, although it is not constant, or the patient could not live, but comes on at intervals only.

A fact of some importance in the clinical history of Barsati is, absence of inflammation characterizing the sores. I. V. S. Meyrick, in a lecture delivered at the Aldershot Military Veterinary School in 1881, said, the disease was "non-inflammatory" in its character; and John Hunter, in 1828, wrote, "True suppuration arises from inflammation, terminating in a disposition to heal, which is not the case with cancer." The sore in Barsati in the early stage is, moreover, usually single, as in cancer, rarely becoming multiple until a late stage, and seldom even then; while the sores in parasitic disease are mostly multiple. The points of junction of skin with mucous membrane, as well as parts exposed to friction, are most frequent sites of Barsati ulceration. Hence the favorite seats of Barsati are seen to be the angles of the mouth and the lips, which are liable to irritation by the bit, &c.; the inner aspect of the fetlocks, more especially in horses given to habitual "brushing"; the prepuce; the lachrymal region, &c. The results of operation have likewise proved most unsatisfactory in the case of Barsati as in that of human cancer. I. V. S. Oliphant || writes: "The state of the system existing in Barsati is not one that indicates

‡Veterinary Journal, July, 1880, p. 18.
||Veterinary Journal, July, 1880.

the use of the knife;" and Dr. Walshe says, "Cancers which have become quiescent have sometimes been cut out, and the operation been followed by rapid reproduction of the disease." Dr. Cooke writes: "From 1851 to the end of 1863, we have had the opportunity of seeing at the Cancer Hospital 413 persons who had been operated on for cancer; and it will astonish the reader to be told that the average lapse of time before the disease returned in these cases was no more than *six and a half months*."* The experience of Veterinary-Surgeons in India shows that Barsati sores which, under appropriate treatment, generally subside about the end of September, re-develop their activity next March, April or May.

We have already shown that the presence of tubercle-like nodules in Bars^ti sores is diagnostic. And Dr. T. Weeden Cooke, on the subject of cancer in man, on page 93, writes: "In many cases the incisions made for the purpose of removing the diseased mass have scarcely healed before '*tubercles*' have appeared around the cicatrix, or the wound itself has taken on the diseased action." Dr. Green† says: "The cells may be so closely packed as ultimately to become hard and dry like those of nails and hair, and the globes are then of a brownish yellow color and of a firm consistence. These globes are often large enough to be readily visible to the naked-eye." Professor Billroth considers the presence of nodules in the skin, when numerous and well-characterised, *diagnostic* of one variety of cancer he has specially studied in Europe.

In their general history, clinical and post-mortem characteristics, course of development, and in the nature and extent of influence producible upon them by treatment, Barsati in the horse and cancer of man are identical. Excepting perhaps the lesser fatality in herbivorous animals noted by M. Leblanc,‡ there is perhaps no feature of Barsati which may not be observed in cases of human cancer. Some of the above facts, I trust, may help accurate diagnosis, for I am of opinion that under the name of Barsati are often included a

*On Cancer: Its allies and counterfeits, pp. 92–3.

†Green, Morbid Pathology, 1878.

‡Recueil de Med, Veterinaire, 4th Series.

host of other diseases which have only a surface or chance resemblance in common. It is the custom at the present day to call every ulcer which is long in healing "Barsati." I trust this will not influence future diagnosis. On the other hand, a simple wound may precede the development of true Barsati by weeks or months. Analogous to this form of disease is epithelioma in the human subject, as a sequel to psoriasis of the tongue. Many veterinarians must, like myself, have met with many cases of Barsati originating in simple sores.

It has been asserted by many persons that the disease is capable of being cured, and, indeed, that it naturally terminates with the rainy season. In this "cured" condition the disease will be found, however, to put in an appearance year after year, when its activity is set on foot; destructive lesions, both external and internal ensue; health is superseded by disease, and death becomes imminent. A most misleading statement was recently made as to the reputed prevalence of Barsati during the rains only. Experience has long since shown that the disease may occur either before, during, or after the rainy season.

The disease successively appears, recedes, recurs, exacerbates—all these under the influence of extrinsic causes, the chief of which is irritation, the result of atmospheric causes, of attacks from flies, and that resulting from the application of corrosive dressings, the use of the knife, &c. In fine, all that is calculated to excite inflammation in the part arouses the disease from its lethargic condition into active play by favoring the extension of the diseased cells in every direction. The latent fault of the system being present, the tendency to form abnormal tissues from imperfectly developed germinal or cellular structure, will be brought into active existence by many external exciting causes. There is no new foreign substance which is deposited among the healthy tissues, but an alteration only of the natural cell-growth or germinating material, which has lost its power of forming healthy structure, results. John Hunter said: "Where schirrus is entirely let alone, it is commonly very slow, and may sometimes continue for years; but when inflammation is excited, its progress is accelerated, inasmuch as inflammation is an increased action of the part itself as well as of the surrounding parts."

It is said that recession of the sores is peculiar to Barsati only, but I am not enabled to endorse this opinion. It is well known that alternate cessations and recurrences of the sores characterize human cancer quite as much as they do Barsati. Dr. Campbell de Morgan, F. R. S., writes*: "A remarkable and not very explicable phenomenon is the arrest of cancer growth and the gradual wasting of the diseased mass. The activity of the whole mass is arrested, new cells cease to be formed, and the tumor fades—*a widely spread mass becoming quiescent and then fading throughout its whole extent.* It shifts the difficulty back a stage or two to suggest that the recession of cancer takes place in obedience to the law under which local atrophy, independent of inflammation or disease, may occur; or that it may be due to some want of organizing power inherent in it from the first, *as some cancers seem born to be atrophic.* It is, under any circumstances, a most important subject for investigation."

From the foregoing remarks it must not be concluded, however, that the period of respite due to atrophic change, is an invariable feature of Barsati. Not by any means has such been found to be the case in our experience of this disease; for, it is usually noticed in the milder cases only, where constitutional symptoms are also absent; whereas it is not at all uncommon to see sores existing before and persisting throughout the winter as well as during the summer. In many cases the disease runs its destructive course in a few months. Had our knowledge of cases ended when the disease first showed signs of local arrestation, we should have been compelled to adopt the prevalent theory of reported cures; but fortunately our cases have gone further, and in too many instances revealed the nature of our panacreas. It is not at all unlikely that some of us have the opportunity of seeing this disease, in our equine patients, at an earlier stage than others generally see it; and sent as these patients are for treatment often after repeated recurrences, we rarely have an opportunity of testing the precise value of many vaunted remedies during its different stages. Experience has shown that recurrence of Barsati

* Med. Times and Gazette, 7 March, 1874, p. 259.

is not confined to the wet weather. It remains to be acknowledged, however, that though this is the case, yet, the disease having once established itself, its return *in loco* is rendered probably more certain during the rains than in any other season. The fact itself, we may observe, appears inexplicable to many who recognize it as such, but its causes are, notwithstanding, capable of explanation; for, as Dr. Moxon* has observed, moisture intervenes, together with the other unfavorable influences of accompanying heat, etc., during wet weather, which augment cell-growth in cancer structures. The effect of moisture in the atmosphere during the rains, therefore, is an increase of cell-activity and a general acceleration of all the worst accompaniments of Barsati.

Many observers exalt special remedies in the treatment of Barsati, and as usual, each produces a list of cures in favor of his favorite application. Further evidence of the difficulty of settling this point is afforded by general experience which disproves the utility of these cures, and shows that their benefits have been overestimated. It is now proved that it is impossible to influence the course of Barsati by recourse to any number of acknowledged parasiticides which have been recommended; and I have myself tried, in numerous cases, local applications of caustic potash, nitrate of silver, chloride of zinc, chlorate of potash,† sulphate of copper, of iron and zinc, salicylate of soda, salicylic,‡ nitric, sulphuric, acetic, and carbolic acids, myrrh, aloes, camphor, alum, sulphur, &c., without effect. Iodine and sulphurous acid injections have been latterly tried by me in many private cases, with equally useless results. T. Marriott, A. V. D., speaks very highly of the results of treatment by iodoform. Very extensive Barsati growths on the fetlocks and lower lip and angles of the mouth have been gradually destroyed by caustics followed by iodoform dressings, and a healthy cicatrix has been obtained. How long an immunity from this disease has been secured, it would be difficult to say; but at any rate it is a considerable

* Moxon, " Trans. Path. Soc.," XX, 28. See Arnott " on Cancer," 1872, p. 70.

† Berl. klin. Wochenschrift, No. 6, 1873.

‡ Wien. Med. Wochenschrift, No. 24, 1883.

gain to have overcome, even for a time, the unsightly and ever increasing out-growth, and to have thus early relieved the animal from pain, and returned him to duty. Calomel and biniodide of mercury were largely employed in the government studs some years ago, but with no better results than any of the above mentioned. Carbolic acid, when well diluted, has the effect of cleaning the surface; but when in strong solution, undoubtedly aggravates the growth in many cases. For the purpose of removing discharges from the surface, and keeping the sore free from smell, I never employ a stronger solution than 1 part in 20 parts of water, for the reasons above stated. Its efficacy, however, is no greater than that of most other agents in common employment. Mr. John Henry Steel, A. V., D. recommends the iodide of arsenic ointment (1–6) as a useful application, to cause a slough. The use of the actual cautery and of the knife is in every case contra-indicated, as it generally increases the activity of the growth. Thus we have tried every remedy, and during every stage of this disease, with the same negative results. At the risk of appearing tautological I would add one other remark upon these operations by the knife, actual cautery and caustic agents. "Be quite sure that you remove *all* the induration, or you will do mischief instead of good." I might give a long list of "successful cases," such as are frequently recorded in the veterinary journals; but as I know from experience such cases have returned for treatment after they had been passed as "cured," I set no value on these temporary successes, and look upon them as mere compromises, which advancing knowledge will enable practitioners to discard, for a more radical and permanently successful treatment. So long as we continue to look upon Barsati as a mere local disease, so long shall we fail to effect more than a temporary recovery.

Where Barsati is indolent, it is wise not to attempt its removal by the knife or by any caustic applications. Such indolence or arrested growth in an external sore is sometimes coincident with its internal devopment. Inspecting Veterinary-Surgeon Oliphant says—"The state of the system existing in Barsati is not such as indicates the use of the knife." Dr. Walshe, after condemning operation for the relief of human cancer, quotes Dr. Macfarlane, who "could adduce the cases of several patients who had labored under cancer for ten, fifteen, and twenty years who were cut off in three or four months by an operation."

*Pari passu* with the advance of the external sore I have noticed defects produced in the constitution of the animal, which should be early supported by appropriate medicinal remedies. The results of this combined treatment will be generally found to be more satisfactory, although how long an immunity from this disease may thus be secured in all cases I am unable to say, but at any rate it is found to be a considerable improvement on the local treatment only, and affords better hope of restoring the patient to work and keeping him out of sick-lines long after others have been admitted with a locally recurrent disease of aggravated character. I know from early experience that it is no good "patching up" a case and returning to duty, which comes back in a few months with considerable constitutional disturbance. I know of no specific remedies that will avert the constitutional derangement, but would recommend the system to be supported on general principles, by the administration of both vegetable and mineral tonics, but especially by iron and arsenic in large doses. Any of the preparations of phosphorus might also prove useful. The influence of phosphorus and arsenic on the general nutrition is analogous to their influence on the nuitrition of the skin and subcutaneous tissues, which is seen in the case of many skin affections.

Our search for agents to neutralize and destroy this disease in its local aspects should lie in the direction of those which shut it up, or encase it, as it were, by hardening its cells The subcutaneous injection of medicine promises the most likely means of doing this. The best agent which will affect this is chromic acid, which in the field of the microscope is seen, even in a solution as dilute as one part in a thousand of water, rapidly to define, as if dissecting out the delicate cells, hardening and enclosing the nuclei, closing up their walls, and so preventing the diffusion of their contents. Professor Billroth's use of this agent as subcutaneous injection in lymphoma of the human subject, gives encouragement to the trial of the same in equine Barsati. Akin to the action of the subcutaneous injection, the use of chloroform in external application bids fair to be useful in the treatment of local Barsati. It is a most useful "carrier" to vegetable alkaloids, promoting their absorption. Its action in causing the absorption of vegetable alkaloids seems to depend upon the removal of the cuticle, thus exposing the absorbing surface of the cutis vera. Professor Trinchera,* of the Naples Veterinary School, believes he has cured cancer of the penis and scrotum in the carnivora, by early operation, the wound being dressed with chlorate of potash. Malthe, reccommends combining iodoform with nitrate of silver as a caustic and healing application in all chronic ulcers. The ulcerated surface is sprinkled with iodoform, on which the nitrate of silver is next applied, and on this again iodoform. A brisk effervescence of nitrous acid and insoluble iodine and chloride of silver results. This may be employed with much benefit in Barsati ulceration of not long duration, and where the subcutaneous tissues have not been deeply implicated.

The above remedies have been described by me in many cases with good results, where constitutional disease was not already far advanced. No one remedy can be expected to produce a cure, and this alone illustrates the absurdity of the dogma, that any "specific" can eradicate a general disease by treating its local manifestations only. We must at present hope for a mere gradual suspension of local disease by treating on general principles.

I have given the above summary with the view of furnishing a general experience in regard to results of different modes of treatment in different cases. Most of the remedies mentioned have been tried by me, as well as other practitioners, in the treatment of this disease. I have, however, to say something further of certain preventives which have not been described before, and which come under the first category in the preceding summary. Those cases in which the sore has apparently been of short duration may be much benefited by a change of climate to the Hills.

* Giornale delle razze degli Animale utili e di Med. Veterinaria, 1875.

Those in which the constitution is severely involved may derive temporary benefit by a similar change. But in some cases the result is disappointment; the local disease persists and grows in spite of all treatment, and sometimes fresh ulceration takes place in an already healed cicatrix, so that the treatment of Barsati must be considered only palliative in kind. When the disease has been suppressed by the above-named means, a relapse is rendered less certain if the animal is kept on a strictly starchy diet.

We are not assured that Barsati is hereditary in its character. Colonel McDougal, Superintendent of Central government studs, tried to show from statistics that it was so. A safe practical conclusion to arrive at is, that all stallions which have suffered from this disease, although temporarily recovered from it, should be castrated, and brood mares similarly affected be disposed of by auction sale; they should not be retained for breeding purposes.

